# Synthesis of Lignosulfonate-Based Dispersants for Application in Concrete Formulations

**DOI:** 10.3390/ma14237388

**Published:** 2021-12-02

**Authors:** Sandra Magina, Ana Barros-Timmons, Dmitry V. Evtuguin

**Affiliations:** CICECO-Aveiro Institute of Materials, Department of Chemistry, University of Aveiro, 3810-193 Aveiro, Portugal; smagina@ua.pt (S.M.); anabarros@ua.pt (A.B.-T.)

**Keywords:** lignosulfonate, concrete, laccase, *Eucalyptus globulus*, polyoxometalate, poly(propylene glycol), poly(ethylene glycol), dispersion

## Abstract

Lignosulfonates (LS) are products from the sulfite pulping process that could be applied as renewable environmentally-friendly polymeric surfactants. Being widely used as plasticizers and water-reducing admixtures in concrete formulations LS compete in the market with petroleum-based superplasticizers, such as naphthalene sulfonate formaldehyde polycondensate (NSF) and copolymer polycarboxylate ethers (PCE). In this work, different chemical modification strategies were used to improve LS performance as dispersants for concrete formulations. One strategy consisted in increasing the molecular weight of LS through different approaches, such as laccase and polyoxometalate-mediated polymerization, glyoxalation, and reversible addition-fragmentation chain transfer (RAFT) polymerization. The other strategy consisted of preparing LS-based non-ionic polymeric dispersants using two different epoxidized oligomer derivatives of poly(ethylene glycol) (PEG) and poly(propylene glycol) (PPG). Modified LS were used to prepare cement pastes, which were examined for their fluidity. Results revealed that the most promising products are PPG-modified LS due to the introduction of PPG chains by reaction with phenolic moieties in LS. The enhanced dispersant efficiency of the ensuing products is probably related not only to electrostatic repulsion caused by the sulfonic ionizable groups in LS but also to steric hindrance phenomena due to the grafted bulky PPG chains.

## 1. Introduction

Lignosulfonates (LS) are sulfonated technical lignins present in spent liquor (SSL) from the sulfite pulping. Worldwide, approximately one million tons of LS are produced annually [1], which makes LS the most abundant type of lignin available in the market on a large scale representing ca. 88% of the global lignin market [2,3,4,5]. Considering the potential applications of LS, besides burning for energy recovery, it is marketed for specialty applications, such as dispersants for concrete formulations, animal feed, paint and oil industries, and agriculture, among others [2,3,5,6]. Most of these applications are due to the good water solubility of LS, which is the reason for the restriction of the world market to these technical lignins [2].

LS are natural polymeric surfactants that contain both hydrophobic (aromatic rings and aliphatic chains) and hydrophilic (sulfonic, carboxyl, and phenolic hydroxyl groups) moieties. Therefore, LS are widely used as concrete water reducers [7,8], coal water slurry dispersants [9,10], oil-well dispersants [11], and pesticide dispersing agents [12]. LS were first introduced as plasticizers and water-reducing admixtures to concrete in the 1930s [13] and nowadays 60 to 90% of LS are used for this purpose. Concrete consists of a composite material made by mixing cement, aggregates, and water, with or without the incorporation of admixtures [14], which are chemicals used to improve the concrete properties such as workability, mechanical performance and durability [15]. Chemical admixtures include, but are not limited to superplasticizers, the effectiveness of which is assessed by the possibility of reducing the water content in the concrete mixture. Therefore, when using plasticizers, a water content reduction of more than 5% by weight is expected, while superplasticizers provide a water content reduction of greater than 12% by weight [15,16]. LS are the most widely used concrete plasticizers, whose plasticizing and water reduction efficiency, however, is limited compared to synthetic superplasticizers [17,18,19,20]. Indeed, LS competes with petroleum-based polymeric superplasticizers, namely naphthalene sulfonate formaldehyde polycondensate (NSF) and a last-generation recently employed copolymer polycarboxylate ethers (PCE) (Figure S1, Supplementary Materials) [15,17]. PCEs are much more efficient than LS giving rise to concrete with superior fluidity or more noticeable water reduction. However, PCEs are often 10 folds more expensive than LS, since the formers are synthetic petroleum-based products and LS are by-products from the sulfite pulp industry. Therefore, the common industrial practice is to mix both LS and PCEs in concrete admixture systems to improve their working and cost-efficiency. Research results indicate that NSF disperses cement particles and reduces attractive inter-particle forces, such as van der Waals forces, by electrostatic repulsion, whereas PCE acts through both electrostatic repulsion and steric hindrance of nonadsorbing side chains [21,22]. On the contrary, LS dispersing and adsorption mechanism is based only on electrostatic repulsion forces, is pH-dependent, and is influenced by the formation of aggregates [23]. Due to the presence of sulphonic groups in LS structure (likewise in NSF), LS can bind to positively charged cement particles, causing them to be electrostatically repelled, recharging their surface, which prevents the fresh cement mixture from agglomerating, i.e., preventing flocculation of cement particles, thus increasing its fluidity [24].

Some strategies can be used to improve LS performance, including increasing their molecular weight whilst preserving their solubility in water by an enzymatic modification treatment [25,26,27]. The enzymatic modification of lignin is considered a convenient method due to its high specificity, mild reaction conditions, lack of undesired by-products, and being environmentally friendly. Two different enzymes are commonly in use, peroxidases and laccases, though laccases are easier to apply in an industrial process than peroxidases because of the widest range of operating conditions and the nature of the oxidant (oxygen vs hydrogen peroxide). Thus, laccases require molecular oxygen, which has higher stability, lower price, and does not decompose spontaneously into radicals (no inactivation of the enzyme) as hydrogen peroxide [25]. Laccases mainly oxidize the phenolic lignin units [28]; however, they are able to oxidize the non-phenolic lignin units in the presence of certain redox mediators [29,30]. Among a variety of mediators, polyoxometalates (POMs), such as K_5_[SiW_11_VO_40_] and H_5_[PMo_10_V_2_O_40_], have shown to be suitable mediators in laccase-mediator systems for oxidative polymerization purposes displaying synergistic behavior when applied in the presence of laccase [31]. Since the adsorption of chemical admixtures on cement particles is related to the type of charged groups and the charge density [32], another strategy to improve LS performance is the introduction of carboxylic groups, for instance, by oxidation via ozonolysis [33]. Another approach reported to improve the dispersant properties of lignin is the preparation of lignin-based non-ionic polymeric dispersants (amphiphiles) from organosolv, kraft (softwood and hardwood) lignins, or LS [34,35,36]. Lignins were reacted with different commercial epoxidized oligomer derivatives and the most promising results were obtained using poly(ethylene)glycol diglycidyl ether (PEGDE) and poly(ethylene)glycol monoglycidyl ether (EPEG) with softwood lignin. The ensuing amphiphilic derivatives containing both hydrophilic and lipophilic moieties displayed enhanced dispersant properties compared with hardwood lignin-based counterparts. Moreover, the softwood lignin-based amphiphilic derivatives displayed even higher cement dispersibility than LS since only half of the amount of softwood lignin-based dispersants was necessary to achieve the same cement dispersibility compared to that of unmodified LS. Furthermore, the chemical modification of lignins with PEG segments has also been reported to yield PEGylated lignins with potential application as thickener in bio-lubricant formulation [37] and bio-based surfactants [38]. Yet, the discussion on the dispersion mechanisms involved is quite limited.

An alternative pathway to modify lignin that has been reported consists of the reaction of lignin with glyoxal in an alkaline medium, resulting in the corresponding ethylol derivative suitable for adhesive applications. Besides glyoxalation, side lignin reactions take place leading to its depolymerization and repolymerization via condensation reactions yielding adducts with interesting structural properties [39,40,41,42,43].

In the last two decades reversible deactivation radical polymerization techniques (RDRP), such as atom transfer radical polymerization (ATRP) [39,40] and reversible addition-fragmentation chain transfer (RAFT) [41], have been employed to produce regular polymers including the modification of lignocellulosic natural polymers. Regarding lignin modification, the grafting of softwood LS and KL with poly(3-sulfopropyl methacrylate) (PSPMA) and with poly(methacrylic acid) (PMAA) via ATRP at pH 11 were reported [42]. The workability of cement pastes prepared using these hybrid polymers as plasticizers was compared with PEGylated lignin analogs and the commercial PCE superplasticizer. The best results approaching the performance of commercial PCE were achieved when using PMAA-grafted LS. It was suggested that the chemical nature of the grafted chains has a strong effect on the dispersant properties of the ensuing material. Adsorption, zeta potential, and intrinsic viscosity were measured for the grafted lignin analogs in order to examine the correlation between lignin and the chemical nature of the grafted chains in the cement dispersion mechanisms. Yet, no straightforward conclusions could be drawn due to the complexity of the system.

The purpose of this study was to enhance the dispersant properties of LS for further application in concrete formulations using different strategies. The first approach consisted of LS polymerization via POM-mediated laccase oxidation complementing our previous work where laccase was used without any mediators [27]. In the second approach, the LS modification was carried out using RAFT polymerization with 2-(dodecylthiocarbonothioylthio)-2-methylpropionic acid (DDMAT) as the RAFT agent. Then, a third approach consisting of LS glyoxalation was examined. Finally, the modification of LS using two different epoxidized oligomer derivatives, poly(ethylene glycol) and poly(propylene glycol) diglycidyl ethers, PEGDE and PPGDE, respectively, was performed. In order to assess the dispersing performance of the samples including the optimum laccase polymerized LS obtained in our previous work [27], the fluidity/workability of cement pastes prepared with the different modified LS was determined and compared with the results obtained for cement pastes prepared with unmodified LS and two different commercial petroleum-based superplasticizers, PCE and NSF.

## 2. Materials and Methods

### 2.1. Materials and Reagents

Industrial sulphite spent liquor (SSL) and thick SSL (THSL), which is concentrated SSL by evaporation, from acidic magnesium-based sulfite pulping of *Eucalyptus globulus* were supplied by Caima Company (Caima-Indústria de Celulose S.A., Constância, Portugal). Lignosulphonates (LS) from SSL and THSL were purified by dialysis against distilled water for 24 h using a partially benzoylated cellulose membrane of 2000 NMWCO (Sigma-Aldrich, Madrid, Spain), freeze-dried, and then kept in a desiccator [43]. Purified LS from thin SSL, hereafter designated as LSF, contained 21.1 wt.% of HSO_3_ groups and 3.0 wt.% of phenolic OH groups, while purified LF from thick SSL, hereafter designated as LSG, contained 17.1 wt.% of HSO_3_ groups and 2.4 wt.% of phenolic OH groups [43].

Laccase Novozym^®^ 51003 (from *Aspergillus oryzea*) was kindly supplied by Novozymes (Bagsvaerd, Denmark) and used without any further purification. 2-(dodecylthiocarbonothioylthio)-2-methylpropionic acid (DDMAT), poly(ethylene glycol) diglycidyl ether (PEGDE, $\bar{M}$_n_~500 g·mol^−1^) and poly(propylene glycol) diglycidyl ether (PPGDE, $\bar{M}$_n_ ~380 g·mol^−1^) were purchased from Sigma-Aldrich (Madrid, Spain) and used without any further purification. Glyoxal solution (40 wt.% in H_2_O) was supplied by Acros (Lisbon, Portugal). All solvents and other reagents were of analytical grade and were purchased from either Acros (Lisbon, Portugal) or Sigma-Aldrich (Madrid, Spain). All solvents for SEC analysis were HPLC grade. See the structure of chemical species used for LS modification in Figure S2 (Supplementary Materials).

Aqueous solution 0.1 M [SiW_11_Mn^III^ (H_2_O)O_39_]^5–^ (hereafter designated as SiW_11_Mn) was prepared from its potassium salt, which was previously synthesized according to previously published methodology [44].

Two superplasticizers, NFS and PCE, were kindly supplied by Sika Company (Sika Portugal-Produtos Construção e Indústria, S.A., Ovar, Portugal). Portland cement type II A-L 42.5R [45,46] from Cimpor Company (Cimpor Indústria de Cimentos, S.A., Souzelas, Portugal) was used to prepare the cement pastes at Sika Company.

### 2.2. Synthesis of Aqueous Solutions of Molybdovanadophosphate POMs

Molybdovanadophosphate POM solutions were synthesized using stoichiometric amounts of MoO_3_, V_2_O_5_, NaH_2_PO_4_·H_2_O, and anhydrous Na_2_CO_3_ [47]. The synthesis of a 0.1 M [PMo_10_V_2_O_40_]^5−^ (hereafter designated as PMo_10_V_2_) solution was carried out as follows: 14.4 g (0.1 mol) of MoO_3_, 1.82 g (0.01 mol) of V_2_O_5,_ and 1.38 g (0.01 mol) of NaH_2_PO_4_. H_2_O were suspended in 35 mL of distilled water in a 250 mL Erlenmeyer flask using a magnetic stirring bar, at room temperature. Then, 5.3 g (0.05 mol) of anhydrous Na_2_CO_3_ was slowly added in small portions to the stirred mixture, causing CO_2_ liberation. A condenser was placed on top of the Erlenmeyer and the mixture was refluxed for 3 h until a deep red translucent solution was obtained. Then the solution was allowed to cool down to room temperature. At this point, 50 mL of a 2 M H_2_SO_4_ solution was added. The final solution was transferred to a glass flask, flushed with N_2_ (g), and kept in the dark.

A similar procedure was carried out for the synthesis of 0.1 M [PMo_11_VO_40_]^4−^ (hereafter designated as PMo_11_V) solution, but in this case, different amounts of MoO_3_ (15.84 g, 0.11 mol) and V_2_O_5_ (0.91 g, 0.005 mol) were used. Both POM solutions were used as such without further purification.

### 2.3. Polyoxometalate (POM)-Mediated Laccase Modification of LS

The laccase oxidative treatment of LSF was performed according to previously reported methodology [27] with the introduction of a certain amount of a selected POM. The procedure consisted in adding 200 μL of a 0.1 M SiW_11_Mn aqueous solution to 10 mL of a 100 g·L^−1^ LSF solution (pH adjusted to 4.3) in a 25 mL jacketed glass reactor equipped with a magnetic stirring bar and a heating circulating water bath, the temperature of which was set at 40 °C. Then laccase (85 U·g^−1^ LS) was added to the solution. The reaction was carried out at 40 °C under continuous pure oxygen bubbling for 60 min. At the end of the reaction, the water-soluble modified LS solution was cooled down using an ice bath and finally stored in the refrigerator (4–6 °C). The same procedure was carried out using the 0.1 M PMo_10_V_2_ and PMo_11_V solutions.

### 2.4. Synthesis of LS-Based Amphiphiles

The procedure consisted in dissolving 12 g of dry LSG in 20 mL of distilled water in a 50 mL two-neck round bottom glass flask equipped with a condenser and a magnetic stirring bar. The LSG solution (pH~4.6) was heated up to 100 °C and then 17 mL of PEGDE was added corresponding to an LS/PEGDE molar ratio of 1:2. The reaction was carried out at 100 °C for 2 h under a nitrogen atmosphere, without the addition of any catalyst. Thereafter, the reaction mixture was cooled down to room temperature and finally stored in the refrigerator. A similar procedure using the same conditions and reagents proportions was carried out but, in this case, 13 mL of PPGDE was added instead of PEGDE.

For SEC analysis and cement paste preparation, samples were kept in the refrigerator (4–6 °C) and then used as such without further treatment/purification step. For chemical characterization, such as FTIR and NMR analyses, samples were purified by dialysis against distilled water for 8 h at room temperature using a partially benzoylated cellulose membrane of 2000 NMWCO (Sigma-Aldrich, Madrid, Spain), freeze-dried and then kept in a desiccator.

### 2.5. Modified LS Characterization

SEC analysis of modified LS samples was carried out using two PL aquagel-OH MIXED 8 μm 300 × 7.5 mm columns protected by a PL aquagel-OH Guard 8 μm pre-column on a PL-GPC 110 system (Polymer Laboratories, Shropshire, UK) equipped with a RI detector. The columns, injection system, and detector were maintained at 36 °C during the analysis. LS and modified LS were dissolved in 0.1 M NaNO_3_ aqueous solution to a concentration of about 10 mg·mL^−1^ (1% *w*/*v*). The eluent (0.1 M aqueous solution of NaNO_3_) was pumped at a flow rate of 0.9 mL·min^−1^. The calibration was performed using pullulan standards (Polymer Laboratories, Shropshire, UK) covering the molecular weight range of 738–48,000 Da.

Purified LS-based products were characterized by Fourier transform mid-infrared spectroscopy (FT-MIR) using an FTIR System Spectrum BX (PerkinElmer, Boston, MA, USA), coupled with a universal ATR sampling accessory, in absorbance mode from 4000 to 500 cm^−1^ with a 4 cm^−1^ resolution. Samples were analyzed as powders, 128 scans were averaged, and all spectra were baseline corrected and normalized (by the min-max normalization technique [48]) for further analysis.

Quantitative ^13^C NMR and ^1^H NMR spectra of purified LS-based amphiphiles were recorded using an ASCENDTM 500 spectrometer (Bruker, Wissembourg, France) operating at 500.16 MHz for proton and at 125.77 MHz for carbon. The ^1^H NMR and ^13^C NMR spectra of modified LS were registered in D_2_O at 298 K using typical sample concentrations of 2.5% for proton and of 25% for carbon spectra. Sodium 3-(trimethylsilyl) propionate-*d_4_* was used as internal standard (δ = 0.00) in proton spectra. The relaxation delay was 3 s and about 200–300 scans were collected (90° pulse). The quantitative carbon NMR spectra were acquired using a 90° pulse, 12 s relaxation delay, and 18,000–20,000 scans were collected. The internal standard used was acetone (δ = 30.89).

Zeta potentials of LS and modified LS aqueous solutions (0.5 g/L) and commercial superplasticizers, NSF and PCE (diluted to 1% *v*/*v*), were measured using a Zeta sizer Nano Series analyzer (Malvern Instruments, Worcestershire, UK). At least six replicates for each sample were determined and the averaged values were reported including the associated deviation error.

### 2.6. Cement Paste Preparation and Flow Table Test

The workability/fluidity of the cement pastes was assessed by the flow table test at Sika Company (Ovar, Portugal) according to the standard procedure EN 12350-5:2009 [49] with some adjustments. The cement paste was prepared as follows: 400 g of water was added to 1 kg of cement, followed by the addition of 10 g of an admixture solution containing 40 wt.% solids content. Various samples were tested as an admixture in the cement formulation, such as unmodified LS, THSL, different modified LS samples, and two commercial superplasticizers, one PCE and one NSF. The selected products are listed in Table 1 including the corresponding description and reference name. The cement paste components were mixed using a laboratory cement paste mixer for 4 min. During this period, the mixing was stopped and the sides of the mixing bowl were scrapped for 20 to 30 s. Finally, a cement paste slurry was obtained. The paste was filtered to break up lumps that may be present and then poured into the truncated flow cone mold on a glass plate/table. Once the cone was lifted, the cement paste collapsed and spread (Figure S3, Supplementary Materials). The paste was allowed to flow on the plate for 30 s. The maximum diameter of the spread was measured; three values were registered and the average value was reported as the fluidity of the cement paste. This first average value was registered at 8 min with the initial time (t = 0 min) corresponding to the beginning of the preparation of the cement paste. The cement paste was collected and kept for further fluidity procedures, one at 30 min and another final one at 60 min.

## 3. Results and Discussion

### 3.1. POM-Mediated Oxidative Polymerization of LS

Polyoxometalates (POMs) can be inorganic mediators in lignin oxidation by laccase when one-electron oxidized lignin molecules are polymerized by radical coupling and the reduced POMs, in turn, are reoxidised by laccase under aerobic conditions [31]. In this study SiW_11_Mn, PMo_11_V and PMo_10_V_2_ were used for the oxidative polymerization of LS in order to increase its M_w_. POM-mediated oxidative polymerization by laccase was compared to LS oxidation with POM or laccase alone for a comparative purpose (Table 2).

As expected from previous studies [25,27], the enzymatic oxidation of LSF without mediator yielded a modified LS (run 2, Table 2) with significantly increased M_w_. Indeed, the M_w_ increased from 3240 to 11,015 Da after a 60 min reaction time without using any external mediators and enzyme load of 85 U·g^−1^ LS. However, when POMs were used as mediators for laccase oxidation of LS, reactions were not as successful as using only laccase, in terms of increase of M_w_. When SiW_11_Mn was used as a mediator, only a slight increase of M_w_ was observed (run 4, Table 2), from 3240 up to 4820 Da, probably due to the synergetic effect reported by Kim and co-workers [31] leading to oxidative polymerization of LS, but not as effective as using laccase alone. In contrast, when PMo_11_V was used as a mediator, the M_w_ decreased (run 6, Table 2) compared to the initial M_w_ value of unmodified LS (sample 1) thus contributing to the LS oxidative depolymeriztion that prevailed over polymerization [50,51,52]. For this reason, the system LSF-PMo_10_V_2_–laccase was not examined. Finally, when each POM was used alone without laccase, only a slight increase of M_w_ was observed when using SiW_11_Mn (run 3) while both PMo_11_V and PMo_10_V_2_ practically did not alter the M_w_ of LS (runs 5 and 7, respectively) suggesting that the LS structure was not affected by these two POMs and probably no significant chemical/structural modification of LS took place.

**Figure 1:**
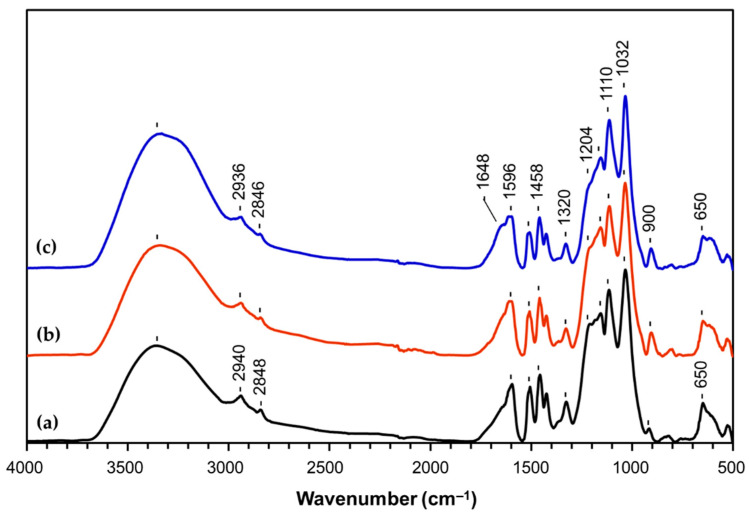
FTIR-ATR spectra of (**a**) LSF, (**b**) SiW_11_Mn-modified LS (LSF-SiW_11_Mn), and (**c**) SiW_11_Mn-mediated laccase-modified LS (LSF-SiW_11_Mn-laccase).

In order to assess the eventual structural changes of LS after modification by POM and POM-mediated laccase oxidative treatment, ATR-FTIR and UV-Vis spectroscopy analyses were carried out to compare LS structure before and after modification with SiW_11_Mn. FTIR-ATR spectra of the initial and modified LS are depicted in Figure 1. The spectra are quite similar except for the weak new band at 1648 cm^−1^ in the spectrum of SiW_11_Mn-mediated laccase-modified LS (LSF-SiW_11_Mn-laccase, Figure 1c) related to the presence of conjugated structures with aromatic double bonds along with an apparent slight reduction of phenolic hydroxyls and methoxyl group content (absorption bands at 1204 and 1110 cm^−1^, respectively) [53,54]. Similar features were already observed in our previous work [27], where laccase was used without any mediator. The similarities between LS and SiW_11_Mn-modified LS (LSF-SiW_11_Mn) spectra suggest that the POM used in the reaction, SiW_11_Mn did not change significantly the chemical structure of LS, probably due to the lack of reactivity.

**Figure 2:**
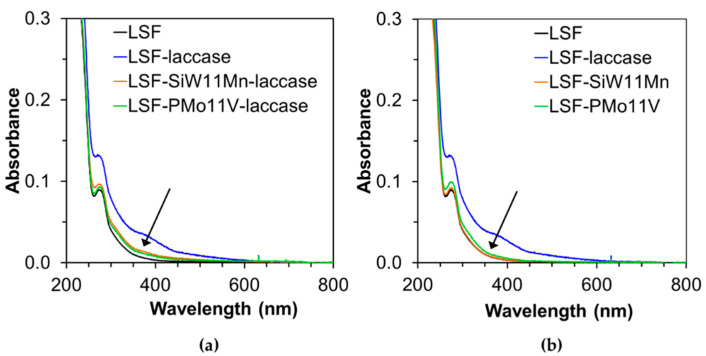
UV-Vis analysis of (**a**) POM-mediated laccase-modified LSF (LSF-POM-laccase), and (**b**) POM-modified LSF (LSF-POM-laccase), where POM is SiW_11_Mn or PMo_11_V. Comparison with unmodified LSF and laccase oxidative polymerized LSF (LSF-laccase). In laccase treatments, laccase load was 85 U·g*^−^*^1^ LS.

The UV-Vis analysis (Figure 2) corroborated the FTIR results. A similar laccase oxidative treatment of LS in the absence of any mediator was carried out for comparison. It is clear that when using only POMs (Figure 2a), the band with a maximum at around 360 nm, attributed to an increased amount of conjugated phenolic structures with α-carbonyl groups or double bonds [55,56], was not observed. Additionally, only a slight increment (almost negligible) of the band around 360 nm was detected (Figure 2b) for the samples obtained after the POM-mediated laccase oxidative polymerization of LS. This clearly corroborates the FTIR analysis: i.e., POMs do not alter the chemical structure of LS and when used as mediators, only impart slight changes to the molecular weight.

Overall, the results obtained showed that, in this case, and considering the reaction conditions, the chosen POMs did not significantly affect the chemical structure of LS even when used as mediators for laccase oxidative modification. Therefore, different approaches were considered to prepare LS-based materials with enhanced dispersant characteristics while preserving the primary structure of LS.

The first alternative method studied was the “grafting-from” approach via RAFT using DDMAT as the RAFT agent. DDMAT, which is a carboxyl-terminated thiocarbonate (Figure S2, Supplementary Materials), has a very high chain-transfer efficiency allowing control over the radical polymerization [57]. Attempts to solubilize DDMAT in water at low pH (around 4) and LS in organic solvents, such as tetrahydrofuran (THF) and pyridine [58,59,60] were unsuccessful. Additionally, other attempts were explored, such as preparing solvent mixtures by dissolving LS in water and DDMAT in other solvents namely THF and dimethyl sulfoxide among others. However, the results were unsatisfactory.

In another approach, the glyoxalation of LS in an alkaline medium was carried out [61], during which hydroxyl groups in ethylol moieties are expected to be introduced in the LS structure. In addition, the eventual depolymerization/repolymerization of lignin via crosslinking yielding adducts with increased *M*_w_ was expected [61,62,63]. Unfortunately, the high pH (>12) necessary to carry out the glyoxalation reaction probably led to changes in the LS structure with the apparent loss of water solubility. As the pH of the LS solution was increased from an initial 4.3 up to 12, by adding NaOH solution, the LS started to precipitate hindering further reaction with water-soluble glyoxal. The precipitation of LS was observed most probably due to the partial desulfonation of LS under these conditions and/or by known reaction of sulfonic groups with aldehyde moieties with the formation of the corresponding semi-acetal adducts favoring the gel formation.

Finally, a third approach was considered, which involved the modification of LS using PEG and PPG derivatives, such as PEG and PPG diglycidyl ethers, PEGDE, and PPGDE, respectively, which is discussed next in detail.

### 3.2. Modification of LS with PEGDE and PPGDE

Grafting PEG moieties onto lignin occurs via nucleophilic substitution through phenolic hydroxide groups usually under moderately alkaline pH conditions (pH > 11) [38]. Previous studies [34,37] reported the successful modification of lignin with epoxidized PEGs in an alkaline medium via epoxy ring-opening reaction. However, in the present study, alkaline pH could not be considered since it compromises the water-solubility of acidic LS due to its partial desulfonization. Nevertheless, the first syntheses were carried out in alkaline medium (with the addition of NaOH until pH~13). However, due to the apparent precipitation of the LS from the solution, this procedure was discontinued. Instead, experiments were then carried out in the water at an initial pH of 2.5 (LS solution) before adding PEGDE or PPGDE. The purified LSG was used in these experiments simulating the industrial thick liquor usually used for the concrete additives.

**Figure 3:**
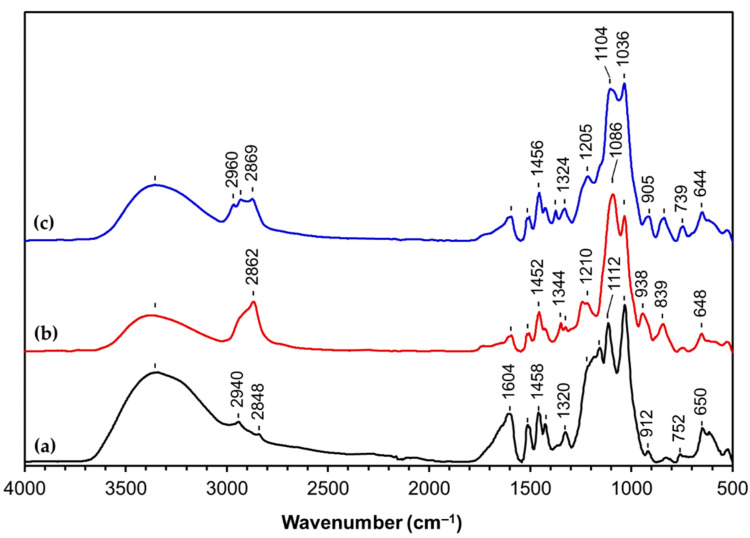
Normalized FTIR-ATR spectra of (**a**) LS, (**b**) LS modified with PPGDE (LS-PPG), and (**c**) LS modified with PEGDE (LS-PEG).

FTIR spectroscopy was used to confirm the structural changes that resulted after LS epoxidation. Figure 3 shows the spectra of LS, LS modified with PEGDE (LS-PEG) and LS modified with PPGDE (LS-PPG). The grafting modification of LS can be confirmed through the presence of a characteristic absorption band at 1250–1210 cm^−1^ assigned to the stretching vibration of C–O–C in the PEGDE structure [64]. Furthermore, the significant decrease in the relative intensity of the OH band around 3350 cm^−1^ in the spectra of LS-PEG and LS-PPG indicates that a part of LS hydroxyl groups was consumed during the modification reaction, which was more pronounced in the case of LS-PPG (Figure 1). The characteristic aromatic bands of LS at 1604, 1510, and 1420 cm^−1^ [53,54,65,66] decreased considerably in LS-PEG and LS-PPG because the final product includes not only LS but also PEG or PPG moieties.

**Figure 4:**
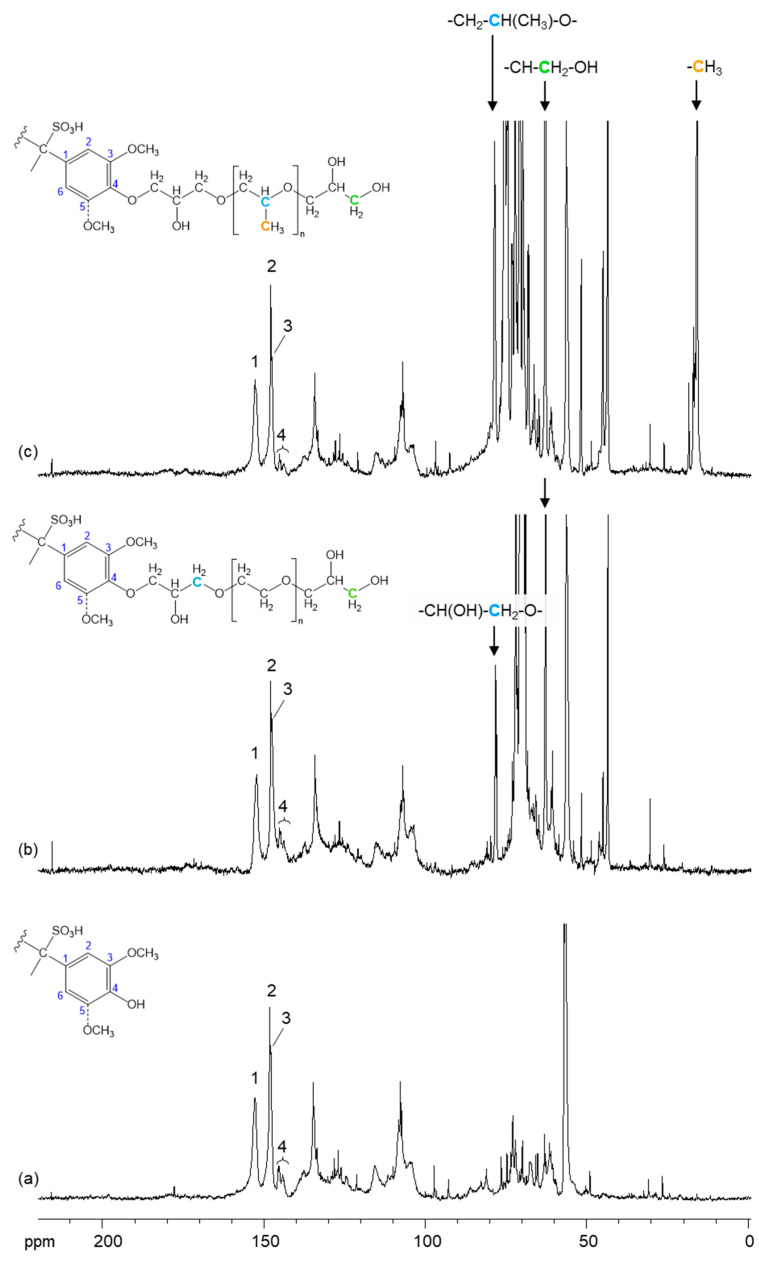
Quantitative ^13^C NMR spectrum of LS (**a**), PEG-modified LS (**b**) and PPG-modified LS (**c**) in D_2_O at 295K.

Additional structural analysis of purified LS, PEG-modified LS (LS-PEG), and PPG-modified LS (LS-PPG) was carried out using quantitative ^13^C NMR and the spectra are presented in Figure 4. Comparing the spectra of LS-PPG and LS-PEG with LS, it is clear that the main differences before and after LS modification occur in the range between 43 and 80 ppm where signals from LS and the PPG/PEG moieties overlap. The expected chemical shifts for the majority of the carbons are quite similar. Therefore, it was not possible to differentiate and identify most of them. Yet, using the ChemNMR ^13^C Prediction tool from ChemDraw Professional software, a few signals from PPG and PEG moieties in each product were identified. Considering the spectrum of PPG-modified LS (Figure 4c and Table 3), the signal at 78.4 ppm is related to the carbon linked to the methyl group (blue carbon in the structure) distributed along the PPG chain. In turn, the signal at 62.7 ppm is related to the carbon linked to the OH group belonging to the terminal group of the PPG chain (green carbon). The signal at 15.2 ppm is assigned to the carbon from the methyl groups in the PPG chain (orange carbon). Analyzing the spectrum of PEG-modified LS (Figure 4b), the signal at 78.1 ppm is assigned to the carbon linked to the oxygen in the PEG chain (blue carbon). In the same way as in the previous spectrum, the signal t 62.7 ppm corresponds to the terminal carbon in the PEG chain (green carbon). Due to the overlapping of signals from LS and PEG/PPG and the fact that most of the carbons in the PEG and PPG are expected to induce identical chemical shifts in the range 72–78 ppm, it was not possible to determine the content of PPG/PEG grafted to the LS structure. Yet, upon the analysis of signals 1, 2, 3, and 4 in all spectra, a subtle difference is observed especially in the intensity of dual signal 4 and unresolved signal 3, which decreased in the spectrum of PPG-modified LS and were assigned to C4 in phenolic guaiacyl (G) and syringyl (S) structures, respectively [67,68,69,70,71,72]. On the contrary, the intensity of signals 1 and 2, which assignments are described in Table 3, did not change so much upon LS modification. The remarked difference in signal intensity, showing in the decrease of intensity of signals 3 and 4 in the spectrum of PPG-modified LS confirmed that PPG-grafting occurred at least via reaction of phenolic hydroxyl groups in G/S structures with epoxy moieties of PPGDE, as illustrated in Figure 4c.

Based on the ^13^C NMR spectra analysis, the content of phenolic OH groups in G structures (signal 4 in Figure 4) and of the methyl groups in the PPG chain was determined and the calculations were carried out per one hundred aromatic rings (100 C6) according to previously established methodology [67,73,74]. The content of phenolic OH groups in G structures present in LS is quite similar to the one in PEG-modified LS, 26 and 28 per 100 C6, respectively. These results suggest that phenolic OH groups did not react or at most reacted to a small extent with PEGDE, which is in agreement with the FITR analysis (Figure 4c). Since phenolic OH groups in syringyl (S) structures are expected to be less reactive than in G structures due to steric hindrance, it is possible that that the reaction of LS with PEGDE resulted only in a negligible modification of LS with no relevant changes in LS structure. Moreover, the molecular weight of PEG-modified LS was similar to that of unmodified LSG (ca. 3500 Da). This indicates that the resonances from the PEG moieties in the spectrum (Figure 4b) probably derive from the PEGDE homopolymer formed during the reaction and that were not removed during the purification by dialysis. Therefore, the product analyzed by FTIR and ^13^C NMR could actually be a mixture mainly composed of LS and PEG homopolymer. On the contrary, the content of phenolic OH groups in G structures present in PPG-modified LS is 15 per 100 C6 against 26 per 100 C6 in LS clearly confirmed that some of these groups reacted with PPG derivative (PPGDE). This is in agreement with FTIR analysis (Figure 3b) where a reduction of the intensity of the band assigned to hydroxyl groups was observed. However, due to the overlapping in the various resonances (Table 3), the accurate calculation of reacted phenolic groups in S structures (signal 3) was impossible. Additionally, the content of methyl groups in the PPG chain is 280 per 100 C6. Even if PPGDE homopolymer was produced during the reaction and was retained in the purified fraction of PPG-modified LS product after dialysis, results clearly corroborate that modification of LS with PPG derivative occurred. In general, it can be concluded that at least half of LS phenolic groups reacted with PPGDE. Regarding the possible reactions of aliphatic hydroxyls of LS, these were discarded due to the very small amount of secondary hydroxyls in LS [68,69] and the practically unchanged intensity of resonance centered at ca. 61.0 ppm (C_γ_H_2_OH in β-O-4′ structures) in the quantitative carbon spectra before and after the reaction with PEGDE/PPGDE (Figure 4).

**Figure 5:**
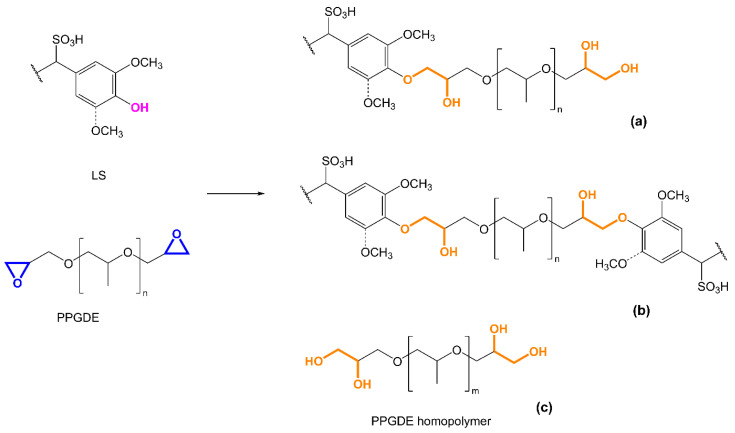
Reaction scheme of LS modification with PPGDE including some possible ensuing products: grafted (**a**) and crosslinked (**b**) derivatives and homopolymer (**c**).

Based on the carbon spectra analysis, the structures of some possible ensuing products derived from the reaction between phenolic groups of LS and PPGDE are depicted in Figure 5, including the structure of the PPGDE homopolymer (Figure 5c). It is possible that PEGDE did not react with LS due to its higher $\bar{M}$_n_ (500 g·mol^−1^) compared to PPGDE ($\bar{M}$_n_ ~380 g·mol^−1^), which may hinder its mobility. Considering this possibility along with the aforementioned steric hindrance of LS aromatic structure, it is possible that epoxide groups in one PEGDE molecule reacted preferably with epoxide groups of another molecule or with water, thus favoring the formation of PEG homopolymer (Figure 5c). In fact, PEGDE has been considered more reactive in homopolymer formation than PPGDE [34,35,36]. In turn, the most probable scenario of LS reaction with PPGDE could be the acid-catalyzed etherification but without crosslinking adjacent molecules (Figure 5a), because the molecular weight of obtained derivatives was increased insignificantly (ca. 3800 Da).

LS are anionic surface-active polymers due to their sulfonic groups. Therefore, LS zeta potential values are highly negative (Table 4). From our previous work [27], laccase-oxidation of LSF resulted in modified LS with increased M_w_, but the content of oxidized functional groups did not change significantly since the zeta potential values remained similar to that of unmodified LSF. In the case of modified LSG, LSG-PEG, and LS-PPG, the reaction did not cause much M_w_ increase of the modified LS and the content of functional groups apparently did not change since the zeta potential values were similar to parent LS. Of particular relevance is the fact that the zeta potentials values of LS and modified LS are quite similar to that of NSF, which was expected since NSF also comprise sulfonic groups (Figure S1, Supplementary Materials). The zeta potential of PCE, however, is −4.7 mV probably due to its lower fraction of ionizable groups per molecule and the larger diffusional path in the electric double layer.

### 3.3. Flow Table Test of Cement Pastes

**Figure 6:**
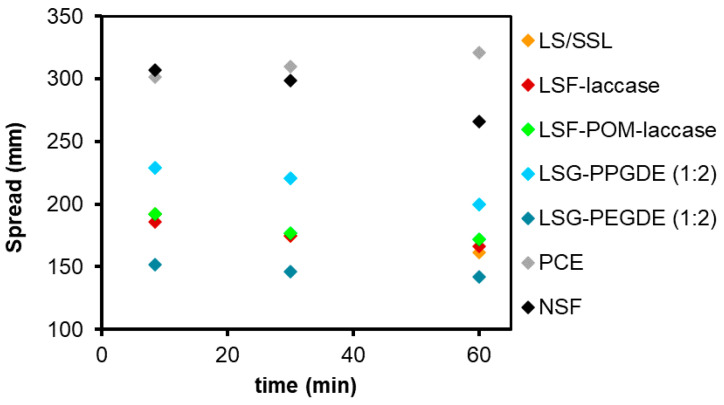
Spread values of cement paste determined by the flow test table (the relative errors did not exceed 5%).

To assess the performance of modified LS regarding the dispersant characteristics for the concrete formulation, the workability/fluidity of cement pastes used in concrete formulations with different admixtures/dispersants (according to Table 1) was studied. The same cement paste formulation was used differing only on the dispersant employed. The flow table test was used for this purpose and the results are depicted in Figure 6. Results obtained from the different modified LS products were compared with those from unmodified LS/SSL (used as a reference and as the minimum value) and with results obtained using two distinct commercial superplasticizers, NSF and PCE.

The results show clearly that the different unmodified LS/SSL, modified LS, and superplasticizers, display different dispersant behavior. Both PCE and NSF yielded the highest cement paste spreading reaching about 300 mm, clearly indicating its superplasticizer ability. Comparing both commercial superplasticizers, the best results were revealed by PCE, especially after 60 min run. This shows that PCE has a retarding effect on the cement paste hardening, allowing higher working time compared to NSF. As already mentioned, the high dispersant efficiency of PCEs is related to both electrostatic repulsion and steric hindrance between their nonionic side chains (Figure S1, Supplementary Materials) [21,22,71], which stretch out into the solution and act as a steric barrier keeping the cement particles at a distance [71,72]. As a consequence, polymeric superplasticizers adsorb on the surface of the cement particle assuming a favorite conformation. This favorite conformation is distorted when a polymer-coated particle approaches another polymer-coated particle. Subsequently, when polymer layers start to overlap, a repulsive steric force emerges [75]. Electrostatic repulsion is related to the surface charge, which arises from the dissociation of surface groups and/or specific adsorption of ions or ionic polymers in water, as well as to the amount of the surface ionizable groups [75].

Noteworthy is the fact that cement pastes prepared with unmodified LS/SSL, laccase-LS, and POM-laccase modified LS products displayed similar fluidity results and were much smaller than those obtained using the commercial superplasticizers (around 190 mm at 8 min and around 169 mm at 60 min). This indicates that laccase oxidative modification of LS with or without any mediator did not affect the dispersant properties of the product. In fact, this is in agreement with the fact that the use of POM for the modification of LS or as the mediator in the laccase modification of LS did not change significantly the structure of LS (Figure 1 and Figure 2). Therefore, a spreading behavior similar to the one obtained using unmodified LS/SSL would be expected.

Gelardi and co-workers [17] have reported that LS comprising a degree of sulfonation up to 0.5–0.7 per phenyl propane unit exhibited optimized water solubility and plasticizing effect as is the case of eucalypt LS used in this study, which contains 0.57 sulphonic groups per phenyl propane unit [43]. However, the results obtained in the present work show that LS dispersant properties are far from comparing to those of commercial superplasticizers. This is due to the fact that the LS dispersant and adsorption mechanism is affected by electrostatic repulsion forces, is pH-dependent, and influenced by the formation of aggregates [23]. Due to the presence of sulphonic groups in LS structure (just like in NSF), LS can bind to the positively charged cement particles, causing electrostatic repulsions, which hinder the agglomeration of the fresh cement mixture, i.e., prevent flocculation of cement particles resulting in an increase of its fluidity [24]. Considering the fact that the fluidity of the cement paste was not significantly enhanced when using LS modified via laccase-oxidative polymerization (with increased *M_w_* of 3,6800 Da [27] but mostly unaltered zeta potential) suggests that the number of ionizable groups did not change much, as proposed by Areskogh and co-workers [27]. Indeed, the increase of the number of sulphonic groups is required to chelate calcium ions hence, modifying the adsorption of LS on the surface of cement particles, by altering the electrostatic repulsion and improving fluidity [24,76,77]. Therefore, this outcome suggests that the sole increase of molecular weight of LS is not crucial or even relevant to enhance the dispersing properties of LS.

The modification of LS with PEGDE or PPGDE yielded products that displayed completely distinct dispersant behavior when tested in cement pastes (Figure 6). PEG-modified LS gave rise to spreading values (152 mm at 8 min and 142 mm at 60 min) quite below those obtained for unmodified LS and SSL (185 mm at 8 min and 161 mm at 60 min). This could be explained by the fact that the product is most probably composed of a mixture of PEG homopolymer and unmodified, or only slightly modified LS, therefore not comprising structural features that could enhance its dispersant properties. In contrast, PPG-modified LF clearly afforded a cement paste with remarkably enhanced fluidity (229 mm at 8 min and 200 mm at 60 min) compared to the cement paste formulated with unmodified LS/SSL (185 mm at 8 min and 161 mm at 60 min). According to the FTIR and ^13^C NMR chemical and structural analyses of this product, PPG chains were grafted to the phenolic moieties in LS as illustrated in Figure 5a. Even with the possible presence of PPG homopolymer, the dispersant properties of this product were undoubtedly improved compared to unmodified LS/SSL probably due to the presence of polyether chains (just like in PCEs) that are responsible for the steric hindrance effect. Consequently, it is possible that the dispersant efficiency of PPG-modified LS is due to electrostatic repulsion (caused by the ionizable groups such as sulfonic groups) combined with steric hindrance (due to the grafted PPG chains). These results show that PPG-modified LS (with an LS:PPGDE molar ratio of 1:2) could be a promising dispersant for concrete formulations.

Based on these promising results, new formulations of PPG-modified LS were prepared with higher PPG content (LS-PPGDE ratios of 1:3 and 1:4). However, the fluidity of cement pastes containing these products was similar to the fluidity of cement pastes comprising unmodified LS/SSL (data not shown). Therefore, it is plausible that the reaction conditions as well as the higher load of PPGDE were favorable to the formation of PPG homopolymer. Even with the modification of LS, the presence of more PPG homopolymer may reverse the dispersant effects of the product, due to the higher concentration of PPG homopolymer in solution, which would probably interfere with the adsorption of PPG-modified LS onto the surface of the cement particles.

## 4. Conclusions

Different chemical modification strategies have been applied to improve LS dispersion efficiency for concrete formulations. The increase of the molecular weight of LS through POM-mediated laccase modification of LS did not significantly change the LS structure. Subsequently, other approaches to LS modification were examined, albeit unsuccessfully in relation to LS glyoxalation and RAFT polymerization grafting. Finally, the synthesis of LS-based non-ionic polymeric dispersants using two different epoxidized oligomer derivatives of PEG and PPG, PEGDE, and PPGDE, respectively, resulted in two distinct outcomes. The structural analysis by FTIR and quantitative ^13^C NMR suggested that PEGDE did not react with LS. In contrast, PPGDE reacted with phenolic LS structures. Approximately half of the phenolic LS units were involved in the reaction, while no evidence of reaction with aliphatic hydroxyls was confirmed. Finally, the PPG-modified LS revealed promising plasticizer properties for the workability/fluidity of cement pastes used in concrete formulations due to their enhanced dispersant efficiency probably related not only to electrostatic repulsion (caused by the sulfonic ionizable groups in LS) but also to steric hindrance phenomena (due to the grafted bulky PPG chains).

## Figures and Tables

**Table 1:** Selected LS-based products and commercial petroleum-based dispersants tested in cement pastes.

Material	Description
THSL	Unmodified thick sulfite spent liquor
LS	Unmodified purified LS from SSL/THSL
LS-laccase	Laccase-modified LSF without mediator *^a^*
LS-POM-laccase	POM-mediated laccase-modified LSF *^b^*
LS-PPGDE (1:2)	PPG-modified LSG (LS:PPGDE = 1:2)
LS-PEGDE (1:2)	PEG-modified LSG (LS:PEGDE = 1:2)
PCE	Commercial superplasticizer
NSF	Commercial superplasticizer

*^a^*—Laccase load of 500 U·g^−1^ LS from previous work [27], *^b^*—Laccase load of 85 U·g^−1^ LS, POM used was SiW_11_Mn.

**Table 2:** Molecular weight of LSF modified by laccase with and without POM (40 °C).

Sample	Material	M_w_ (Da) 0 min	M_w_ (Da) 60 min
1	LSF	3240	‒
2	LSF‒laccase *	‒	11,015
3	LSF-SiW_11_Mn	‒	4500
4	LSF-SiW_11_Mn-laccase *	‒	4820
5	LSF-PMo_11_V	‒	3430
6	LSF-PMo_11_V-laccase *	‒	2880
7	LSF-PMo_10_V_2_	‒	3300

*—Laccase loads of 85 U.g^−1^ LS.

**Table 3:** Assignment of carbon signals in ^13^C NMR spectra of LS and modified LS [67,68,69,70,73,74].

Signal	δC (ppm)	Assignment
LS, PEG-modified LS and PPG-modified LS (all spectra)		
1	152.8	C3,5 in etherified S structures
2	148.1	C3 and C4 in etherified G, C4 in etherified S structures
3	148.0	C3,5 and C4 in phenolic S structures
4	145−146	C4 in phenolic G structures and tannins
PEG-modified LS		
-	78.4	-CH_2_-**C**H_2_-O-
-	62.7	-CH(OH)-**C**H_2_-OH
PPG-modified LS		
-	78.4	-CH_2_-**C**H(CH_3_)-O-
-	62.7	-CH(OH)-**C**H_2_-OH
-	15.2	-**C**H_3_

**Table 4:** Zeta potential values of unmodified LS and modified LS by laccase oxidation and modification with PEGDE and PPGDE.

Material	C (g/L)	pH	Zeta Potential (mV)	Reference
LSF	0.51	4.4	−25.6 (±0.9)	[27]
LSF-laccase (83) *	0.51	4.3	−24.9 (±0.9)	[27]
LSF-laccase (250) *	0.51	4.5	−24.0 (±0.5)	[27]
LSF-laccase (500 *) *	0.51	4.6	−23.4 (±0.7)	[27]
LSG	0.51	4.6	−20.9 (±1.1)	–
LSG-PPG	0.57	6.3	−21.1 (±0.8)	–
LSG-PEG	0.61	6.3	−19.9 (±0.9)	–
NSF	1% *v*/*v*	7.0	−29.3 (±3.4)	–
PCE	1% *v*/*v*	5.3	−4.7 (±1.6)	–

* Laccase loads of 83, 250 and 500 U g^−1^ of LS.

## Data Availability

Data sharing not applicable.

## Supplementary Materials

The following are available online at https://www.mdpi.com/article/10.3390/ma14237388/s1, Figure S1: Chemical structure of superplasticizers: NSF (a) and example of PCE (b); molecular architecture of a comb-like PCE superplasticizer (c), Figure S2. Structure of chemical species used in this study for LS modification: RAFT agent, DDMAT (a), glyoxal (b) and epoxidized oligomers derivatives, PEGDE and PPGDE (c), Figure S3: Flow table test: spreading of cement paste.
