# Supplementary material for: Synthesis of Lignosulfonate-Based Dispersants for Application in Concrete Formulations
Source: Materials (Basel). 2021 Dec 2;14(23):7388. doi: 10.3390/ma14237388 (PMC8658405; doi:10.3390/ma14237388)
Supplement: Supplementary file 1 [file materials-14-07388-s001.zip › materials-1463448-supplementary.pdf]

materials

Supplementary Materials

Synthesis of Lignosulfonate-Based Dispersants for Application in Concrete Formulations

Sandra Magina, Ana Barros-Timmons, Dmitry V. Evtuguin*

CICECO-Aveiro Institute of Materials and Chemistry Department, University of Aveiro, Campus de Santiago, P-3810-193 Aveiro, Portugal

** Corresponding author: Dmitry V. Evtuguin (e-mail: dmitrye@ua.pt)*

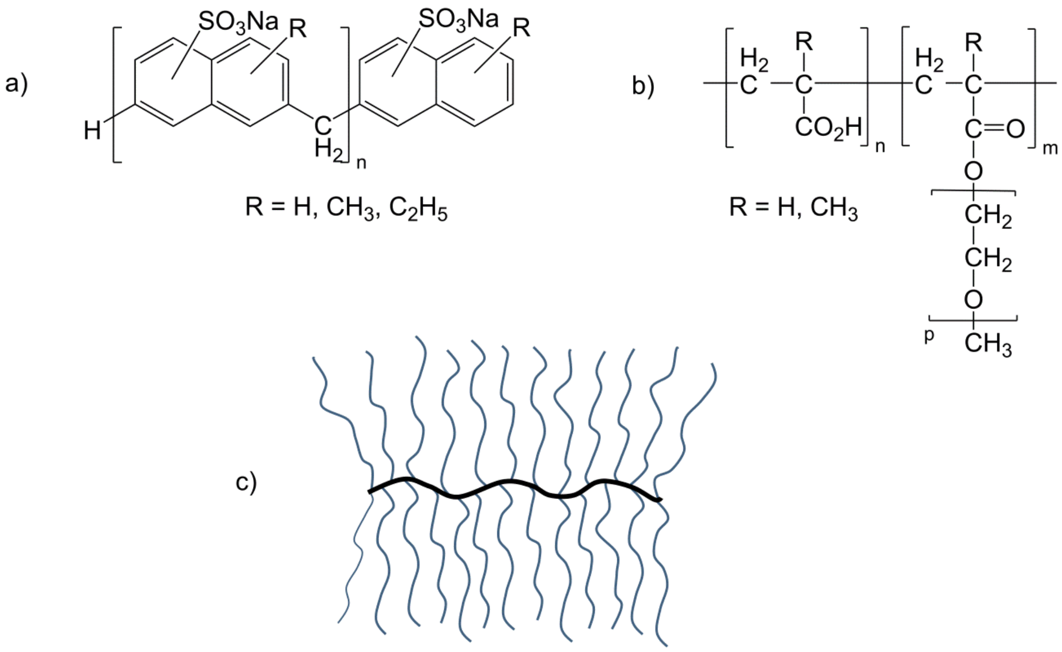

Figure S1. Chemical structure of superplasticizers: NSF (a) and example of PCE (b); molecular architecture of a comb-like PCE superplasticizer (c).

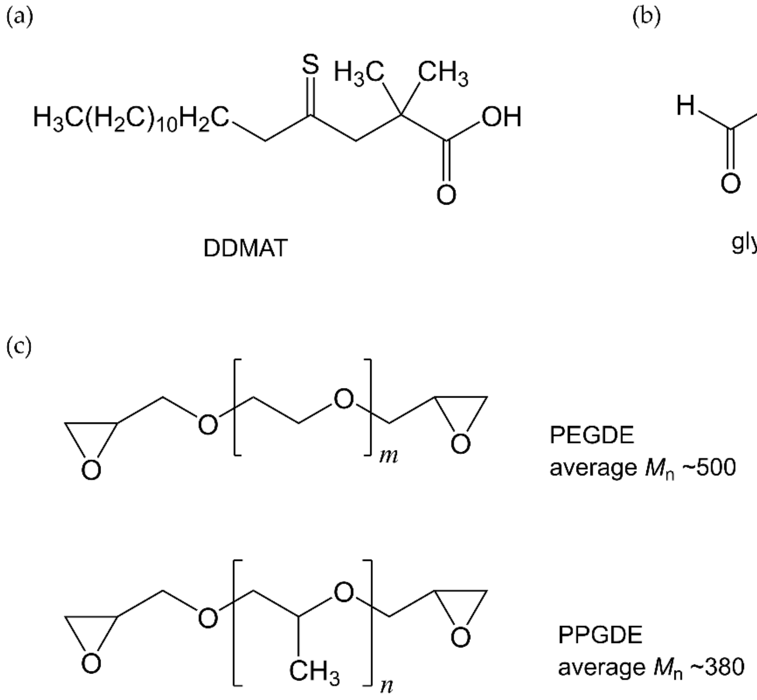

Figure S2. Structure of chemical species used in this study for LS modification: RAFT agent, DDMAT (a), glyoxal (b) and epoxidized oligomers derivatives, PEGDE and PPGDE (c).

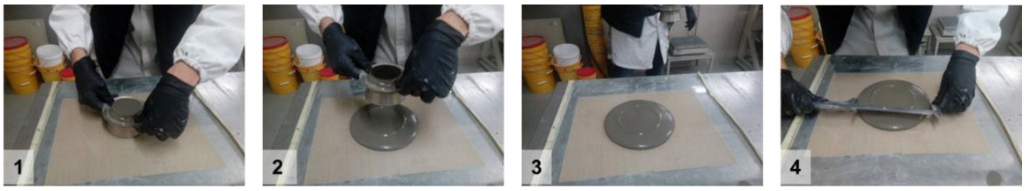

Figure S3. Flow table test: spreading of cement paste.
